# *Serratia marcescens* in the intestine of housefly larvae inhibits host growth by interfering with gut microbiota

**DOI:** 10.1186/s13071-023-05781-6

**Published:** 2023-06-10

**Authors:** Ying Li, Shumin Wang, Kexin Zhang, Yansong Yin, Xinyu Zhang, Qian Zhang, Xinxin Kong, Luyao Tang, Ruiling Zhang, Zhong Zhang

**Affiliations:** 1 School of Basic Medical Science, (Shandong Academy of Medical Sciences), Shandong First Medical University, Taian, 271016 Shandong China; 2 Collaborative Innovation Center for the Origin and Control of Emerging Infectious Diseases, (Shandong Academy of Medical Sciences), Shandong First Medical University, No. 619, Changchen Road, Taian, 271016 Shandong China; 3 School of Life Science, (Shandong Academy of Medical Sciences), Shandong First Medical University, Taian, 271016 Shandong China; 4 Weifang Medical University, Weifang, 261021 Shandong China; 5 The First Affiliated Hospital of Shandong First Medical University, Jinan, 250014 Shandong China

**Keywords:** Housefly larva, Gut microbiota, *Serratia marcescens*, Bacteriophage, Microbiota–host interaction, 16S rRNA gene sequencing

## Abstract

**Background:**

The structure of gut microbiota is highly complex. Insects have ubiquitous associations with intestinal symbiotic bacteria, which play essential roles. Thus, understanding how changes in the abundance of a single bacterium interfere with bacterial interactions in the insect’s gut is important.

**Methods:**

Here, we analyzed the effects of *Serratia marcescens* on the growth and development of housefly larvae using phage technology. We used 16S rRNA gene sequencing technology to explore dynamic diversity and variation in gut bacterial communities and performed plate confrontation assays to study the interaction between *S. marcescens* and intestinal microorganisms. Furthermore, we performed phenoloxidase activity assay, crawling assay, and trypan blue staining to explore the negative effects of *S. marcescens* on housefly larvae’s humoral immunity, motility, and intestinal organization.

**Results:**

The growth and development of housefly larvae were inhibited after feeding on *S. marcescens*, and their intestinal bacterial composition changed with increasing abundance of *Providencia* and decreasing abundance of *Enterobacter* and *Klebsiella*. Meanwhile, the depletion of *S. marcescens* by phages promoted the reproduction of beneficial bacteria.

**Conclusions:**

In our study, using phage as a tool to regulate the abundance of *S. marcescens*, we highlighted the mechanism by which *S. marcescens* inhibits the growth and development of housefly larvae and illustrated the importance of intestinal flora for larval development. Furthermore, by studying the dynamic diversity and variation in gut bacterial communities, we improved our understanding of the possible relationship between the gut microbiome and housefly larvae when houseflies are invaded by exogenous pathogenic bacteria.

**Graphical Abstract:**

**Figure:**
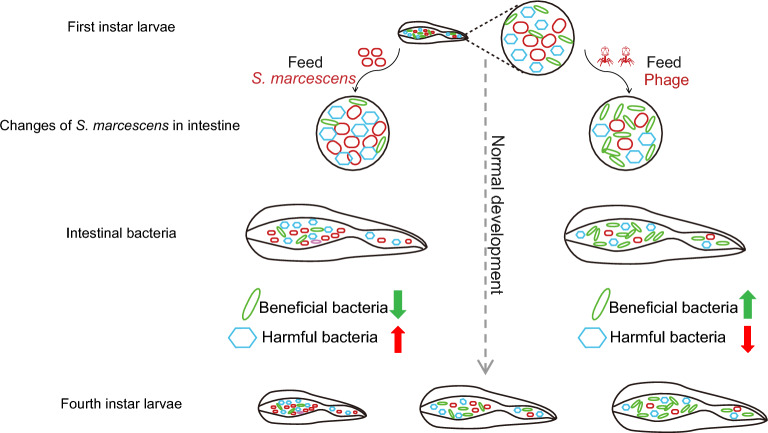

**Supplementary Information:**

The online version contains supplementary material available at 10.1186/s13071-023-05781-6.

## Background

The gut microbiota includes bacteria, archaea, viruses, and fungi [1, 2] and affects the health of housefly larvae in various ways [3]. The composition of the housefly microbiota depends on the insect’s intestinal environment [4], and bacteria in the microbiota influence the daily activities of housefly larvae via physiological functions, including nutrition, metabolism, development, behavior, and immunity [2, 3]. Gut microbiota alterations can have beneficial effects by stimulating hosts and immune mechanisms, thereby conferring resistance to pathogenic invasion [5]. Conversely, an imbalanced or depleted microbiome may be harmful to insects, leading to various diseases [6]. Previous studies reported that culturable bacteria in the housefly larvae gut strongly influence the insect’s growth and development, humoral immunity, and intestinal microbiota diversity.

An example of such effects is *Rhagoletis pomonella* (Diptera: Tephritidae), whose gut bacteria can consume and digest plant-derived toxic compounds, providing constant nutrition for the host [7]. Moreover, in the Mediterranean fruit fly (*Ceratitis capitata*), the *Enterobacteriaceae* community can contribute to the fly’s development and copulatory success, extending the fly’s lifespan and conferring resistance to deleterious bacteria [8]. Meanwhile, when *Enterobacter hormaechei *was added to the feed of housefly larvae as a beneficial bacterium, the growth of some pathogenic bacteria in the intestine was inhibited, which accelerated the growth and development of housefly larvae [9]. Furthermore, *Pseudomonas aeruginosa* Y12, “harmful bacterium” within the housefly larvae’s gut, contributed to its host fitness by producing antifungal compounds as a form of defense against *Beauveria bassiana* [10], although high concentrations of *P. aeruginosa* Y12 had adverse effects on the health of housefly larvae, which affected the bacterial communities in their gut [11]. Additionally, we previously investigated the dynamic effects of phages on a model microbiome [12, 13]. Upon creating an intestinal phage amplification model by multiple or single administration of bacteriophages, changes were induced in the intestinal bacterial composition, which consequently led to further changes in the health of housefly larvae. Briefly, these results suggest the potential impact of gut bacteria on their insect hosts, which has implications for their beneficial use to shape the normal gut microbiota composition.

*Serratia marcescens* is a gram-negative opportunistic pathogen infectious to humans and insects. *Serratia marcescens* has been found to facilitate the infection of mosquitoes by an arbovirus, which is thought to be mediated by a protein named SmEnhancin (secreted by *S. marcescens*), as it is known to digest gut membrane-bound mucins [14]. *Serratia marcescens* VA (Vectopole Amazonien) can efficiently colonize *Anopheles* and *Aedes* mosquitoes, in which it has some larvicidal activity [15]. *Serratia marcescens* strains isolated from the gut of bees, when ingested and injected into the body cavity, can lead to lethal infections [16]. However, it remains unknown whether *S. marcescens* can lead to gut dysbiosis in housefly larvae.

Bacteriophages have begun to attract the attention of researchers and have shown therapeutic potential in insects [17]. When their total estimated number is 10 times more than that of their bacterial prey/host, they are considered an effective tool for shaping intestinal bacterial communities [18, 19]. As specific bacterial predators, bacteriophages (viruses that specifically infect bacteria, not fungi or other microorganisms) can change microbial diversity through Red Queen/kill-the-winner dynamics [20, 21]. Phages are the most common and widely distributed group of viruses [22], and there is increasing evidence that bacteriophages play an important role in regulating microbial ecosystems [23, 24]. The pathogen-specific viruses used in antibacterial strategies provide an accurate species-specific mechanism for infecting host bacteria [25]. Phages can be considered a tool to precisely target and modulate susceptible species in the gut, leaving the untargeted microbiota unaffected [26, 27].

To study the effects of *S. marcescens* on the growth of housefly larvae and microbiota–host interactions, we used housefly larvae, *S. marcescens*, and phage as an insect model of gut-associated bacteria–host interactions. We isolated *S. marcescens* from the gut of housefly larvae and isolated phages that target *S. marcescens* from environmental water. We analyzed the negative effects of *S. marcescens* on the housefly larvae and the mechanisms involved via a bacterial feeding test. Additionally, using phage technology, we analyzed the inhibitory effect of phage on *S. marcescens* via a phage-feeding test and determined the growth and developmental level of housefly larvae after phage depletion of pathogenic *S. marcescens*. To further analyze whether bacterial infection and phage predation changed the composition of the host gut microbiota, we tracked the dynamic changes in gut microbiota using 16S ribosomal RNA (rRNA) gene sequencing. Our work provides insight into how changes in the abundance of a single bacterial species can interfere with interbacterial interactions in the insect intestine. We also explored the changes in the innate immunity of housefly larvae and proved the negative effects of bacterial invasion in housefly larvae by observing intestinal damage and the motor ability of housefly larvae.

## Methods

### Microbial strains and experimental conditions

The housefly colony has been reared in the Laboratory of Vector and Vector-borne Diseases of Shandong First Medical University since 2005.

Using traditional isolation and culture methods, *S. marcescens* were isolated from housefly larval gut and cultured at 37 °C in lysogeny broth (LB) liquid media as described in our previous research [28].

### Bacteriophage isolation

The bacterial strain *S. marcescens* was used as host bacteria to screen bacteriophages as described previously [12, 29]. The isolated phage was named SMP (Additional file 1: Table S1). The high-concentration phage stocks were stored at 4 °C and the phage morphology was observed by an electron microscope.

### Whole-genome and phylogenetic tree analysis

Whole genome sequencing was conducted on the Illumina platform. Phage sequences were deposited in the National Center for Biotechnology Information (NCBI) (accession number: OP490597).

For the single protein-based phylogenetic analysis, the phage terminase large subunit protein sequences were aligned with ClustalW in MEGA 6.0. A phylogenetic tree was constructed in MEGA 6.0 using the neighbor-joining method with 1000 bootstrap replicates [30].

### Phage characterization

A spot test was used to confirm the host specificity of phage SMP [31]. First, 10 μl of 10^8^ PFU/ml phage SMP was dropped on a double-layer agar (0.7% agar) plate containing bacterial solution (*S. marcescens*, *Providencia stuartii*, *Providencia vermicola*, *Klebsiella pneumonia*, *Enterobacter hormaechei* and *Enterobacter cloacae, Acinetobacter bereziniae*, *Lysinibacillus fusiformis*, *Pseudomonas aeruginosa*, *Lactococcus lactis* and *Bacillus safensis*), and plates were incubated overnight at 37 °C. The presence of plaque indicated the lytic activity of phage SMP. The one-step growth curve experiment was performed to determine the latent time and burst size of phage SMP [32, 33]. The stability of phage SMP under different pH (2, 3, 4, 5, 6, 7, 8, 9, 10, 11, 12) and temperature (−80 °C, 4 °C, 25 °C, 37 °C, 50 °C, 60 °C, 70 °C and 80 °C) were determined by standard methods [12]. Phages were diluted into 10^7^ PFU/ml and incubated at specific pH and temperature for 2 h and 1 h respectively, and the double-layer agar plate method was used to detect the titer of the phages. All analyses were performed in triplicate.

### Bacteria and phage infection in a housefly larval model

For bacteria and phage perturbation experiments, 1-day-old housefly larvae were selected for the 5-day feeding experiment. The sterile water and sterilized wheat bran were mixed at appropriate proportions for a final diet. Housefly larvae in different groups were fed sterile water containing bacteria (10^9^ colony-forming units [CFU]/ml) and bacteriophages (10^7^ and 10^11^ plaque-forming units [PFU]/ml), designated as SM, SMPa, and SMPb. The Wa group was the control group that did not contain any bacteria and phages. Three perforated test tubes were used in each group. Five housefly larvae samples were taken from each group of test tubes every day to measure their weight and length until the fifth day. At the end of the experiment, the pupal weight, pupation rate, and emergence rate were analyzed. Larval samples were collected in different test tubes every day for 16S rRNA high-throughput sequencing [12, 13].

### Plate confrontation assay

To determine the interactions between *S. marcescens* and the other housefly gut bacteria (*P. stuartii*, *P. vermicola*, *K. pneumonia*, *E. hormaechei*, *E. cloacae*, *A. bereziniae*, *L. fusiformis*, *P. aeruginosa*, *L. lactis*, and *B. safensis*), we conducted plate confrontation experiments on nutrient agar (NA) medium plates. The plate was divided into two equal parts; half was used for inoculation of *S. marcescens*, and the other half was used as a negative control. Six-millimeter-diameter sterile filter papers were placed on both sides of the agar plate, and 10 μl of *P. stuartii* bacterial liquid was added to the filter paper. Other bacteria were tested using the same method as described above. All the plates were incubated at 37 °C. The growth diameter of each bacterium was measured after 24 h. The experiments were conducted with six independent biological replications.

### Effects of feeding isolated bacteria and phage on phenoloxidase activity in housefly larvae

The feeding experiment for housefly larvae was conducted for 5 days, and larvae samples in each group were collected every day. The housefly larva sample was placed in a centrifuge tube containing phosphate buffer (pH = 7.0) and homogenized. The supernatant was extracted after centrifugation at 4 °C and 12000 r/min for 20 min. The enzymatic reaction system was prepared using the same method as described previously [5, 34]. After reacting in a 25 °C water bath for 15 min, the OD_405_ value was measured.

### Effects of feeding isolated bacteria and phage on intestinal tissue in housefly larvae

According to the reported scheme, trypan blue staining was performed at the ages of 2, 3, and 4 days [35]. Larvae were collected and washed with phosphate-buffered saline (PBS; 1×) solution to remove any food traces. The larvae were then transferred to trypan blue stain [36] and kept under shaking conditions for 30 min. After 15 min, the larvae were thoroughly cleaned with PBS solution to remove any additional dye that may have existed on their surface. The larvae were then observed under a stereoscopic microscope and imaged for cell damage checking .

### Effects of feeding isolated bacteria and phages on motility in housefly larvae

Larvae crawling behavior was monitored by following a previously reported protocol [37, 38]. On the fourth day after feeding *S. marcescens* and phages, three larvae were taken from different groups, placed on crawling trace medium at room temperature, and allowed to crawl freely on the culture medium. After 15 min, photos were taken to record the crawling traces of housefly larvae, which were then marked with Digitizer^®^ 4 to calculate their crawling traces.

### Statistical analysis

All data were analyzed using SPSS Statistics 20 and GraphPad Prism (8.0.2). All data are expressed as mean ± standard deviation. The changes in the body weight and body length of housefly larvae under different treatments were compared using two-way analysis of variance (ANOVA) followed by Šidák correction. The antagonism experiment was analyzed by the *t*-test, and the activity of phenoloxidase in hemolymph of the larvae was analyzed by *t*-test. Asterisks indicate significant difference at *P < 0.05, **P < 0.01, ***P < 0.001, and ****P < 0.0001.

## Results

### Isolation of housefly larvae *S. marcescens* and its associated bacteriophages

**Fig. 1:**
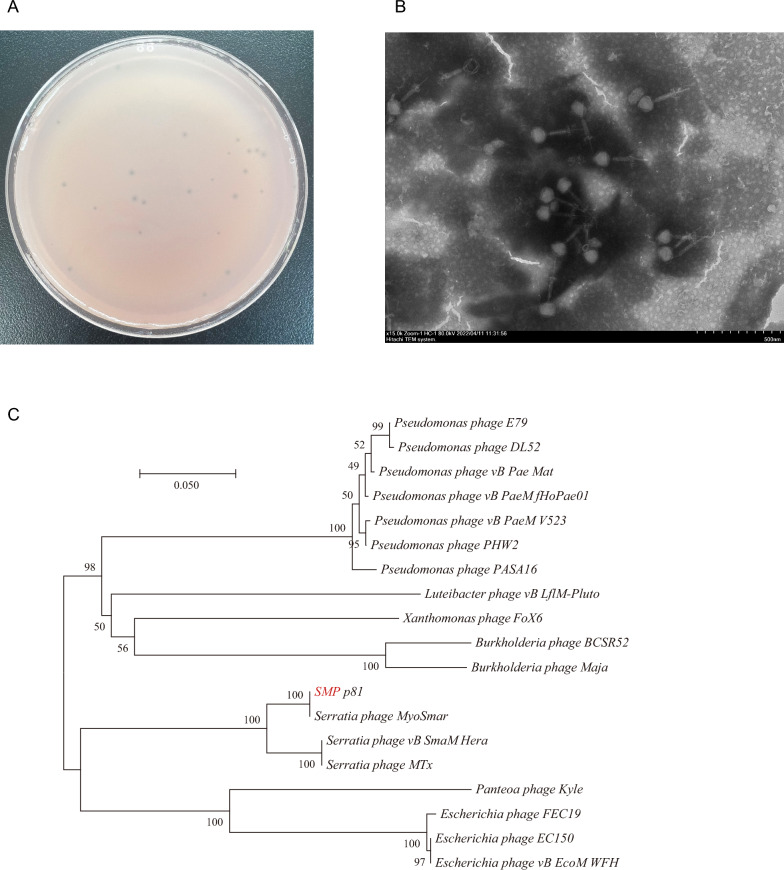
Biological properties of *S. marcescens* phage SMP. **A** Morphology of SMP on agar containing *S. marcescens*. **B** Electron micrograph of phage SMP. **C** Phylogenetic tree based on terminase large subunit proteins to comparative similarity. The phage SMP used in this experiment is marked in red, and data for other similar phages are publicly available from the NCBI. The scale bar represents 0.050 nucleotide substitution percentage, and values next to the nodes show 1000 bootstrap replications

*Serratia marcescens* was obtained by a conventional isolation and culture method from the gut of housefly larvae. The bacteriophage SMP was enriched and isolated from environmental water (Additional file 1: Table S1) using the double-agar-layer method. SMP can effectively lyse *S. marcescens* (Fig. 1A). Transmission electron microscopy showed that SMP belongs to the *Myoviridae* family, with an icosahedral head and a non-contractible tail (Fig. 1B).

The genome of the phage SMP (GenBank accession: OP490597) was found to share a high similarity (98.9%) at the DNA level with the *Serratia* phage MyoSmar (GenBank accession: NC_048800.1). Genome sequencing results showed that the genome of SMP was 68,405 base pairs in size and had a GC (guanine-cytosine) content of 49.19% (Additional file 3: Fig. S1). A phylogenetic tree of the phage SMP based on terminase large subunit proteins was constructed (Fig. 1C).

The host specificity of the phage SMP was determined using a spot test, which revealed that this phage could produce a lytic zone against *S. marcescens*. However, it exhibited no lytic activity against other culturable bacteria in the gut of housefly larvae (Additional file 2: Table S2).

**Fig. 2:**
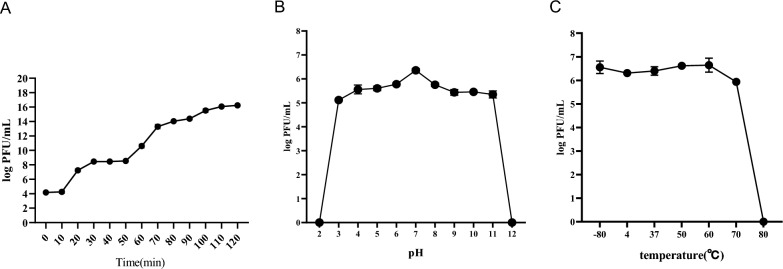
Biological properties of *S. marcescens* phage SMP. **A** One-step growth curve of phage SMP. Phage SMP has a short latent period. The phage titer increased rapidly and stabilized at 100 min. **B** The effect of pH on the stability of phage SMP was determined at different pH values ranging from 3 to 11 at 37 °C for 2 h. **C** Effect of temperature on phage SMP stability was determined at −80 °C, 4 °C, 25 °C, 37 °C, 50 °C, 60 °C, 70 °C, and 80 °C for 1 h. Values are the means  ±  standard deviations from triplicates of each treatment

One-step growth curves showed that the latent period of the phage SMP was approximately 10 min, while the maximum virus yield per cell was about 2.15 × 10^7^ (Fig. 2A). SMP also showed excellent stability, withstanding not only 2 h at pH 3–11 (Fig. 2B) but also 1 h in the temperature range of – 80–70 °C (Fig. 2C).

### Effects of *S. marcescens* and bacteriophage expansion on the growth and development of housefly larvae

**Fig. 3:**
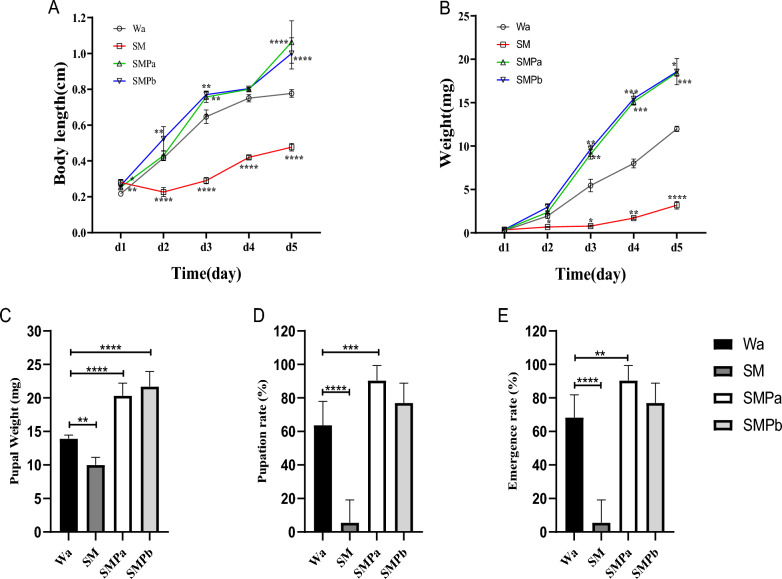
Effects of different treatments on the growth and development of housefly larvae. **A** Changes in the body lengths of housefly larvae in each group. **B** Changes in the body weights of housefly larvae in each group. Impact of different treatments on the **C** pupal weight, **D** pupation rate, and **E** emergence rate of housefly larvae. Wa, SM, SMPa, and SMPb represent housefly larval samples treated with sterile water and 10^9^ CFU/ml *S. marcescens* and 10^7^ and 10^11^ PFU/ml phage, respectively. The data are expressed as the mean ± standard error of the mean (SEM). Repeated-measures ANOVA followed by Šidák correction was used for multiple comparisons. *P < 0.05, **P < 0.01, ***P < 0.001, ****P < 0.0001

To analyze whether *S. marcescens* and bacteriophages can affect housefly larvae, ~ 10^9^ CFU/ml *S. marcescens* and ~ 10^7^ or ~ 10^11^ PFU/ml *S. marcescens*-specific bacteriophage SMP were added to the basal diet of housefly larvae, and the growth of housefly larvae was monitored through body weight and length. On the second day of feeding, compared with that of the Wa group, the growth of housefly larvae treated with *S. marcescens* began to slow, and their body length and weight were only half of those of the Wa group (Fig. 3A, B). Serratia* marcescens* infection significantly reduced the pupa weight, pupation rate, and emergence rate of housefly larvae (Figs. 3C–E). The body weight of housefly larvae fed ~ 10^7^ and ~ 10^11^ PFU/ml *S. marcescens*-specific bacteriophage SMP (SMPa/SMPb group) was significantly higher than the body weight of those in the Wa group, suggesting that bacteriophages had a positive effect on the health of housefly larvae (Fig. 3A, B). These results indicated that *S. marcescens* inhibited the growth and development of housefly larvae and that the increase in the number of bacteriophages in the intestine of housefly larvae promoted their growth.

### Effects of oral intake of *S. marcescens* and phage on intestinal tissues in housefly larvae

**Fig. 4:**
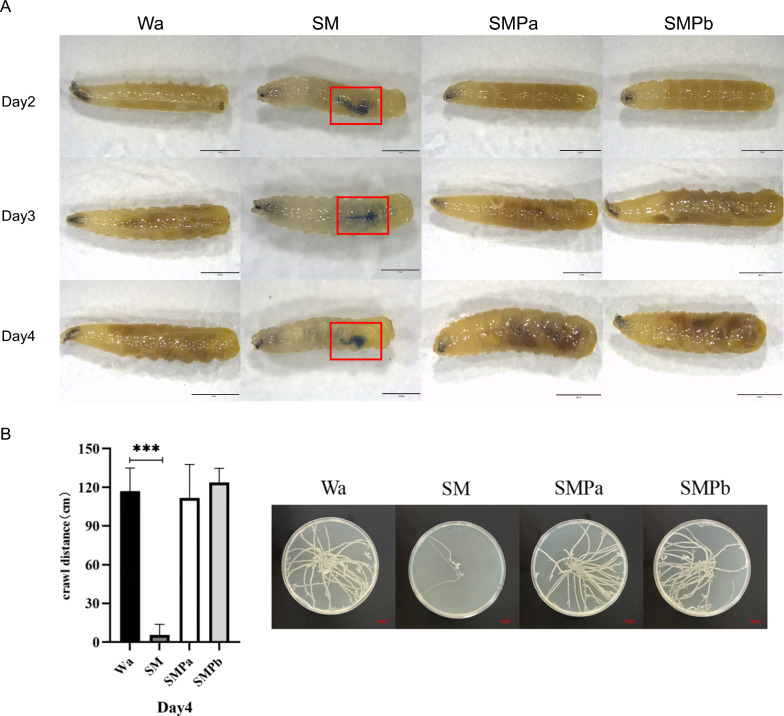
**A** Intestinal damage of housefly larvae. The larvae in the SM group showed blue color due to intestinal damage (red frame). **B** Crawl trace of housefly larvae on the crawl trace culture medium. Values are the means  ±  standard deviations from triplicates of each treatment. *** P < 0.001

To better understand the mechanism behind the damage to larvae caused by *S. marcescens*, we conducted histological analysis (Fig. 4A). Upon comparison with the SMPa/SMPb and Wa groups, the trypan blue staining image of the larvae fed *S. marcescens* showed a clear blue intestinal tract, indicating that *S. marcescens* directly affected the development of the housefly larvae by damaging their intestinal tissues.

### Effects of oral intake of *S. marcescens* and phage on housefly larva motility

Digitizer^®^ 4 was used to label crawling traces on media after 15 min (Fig. 4B). The crawling trace experiment showed that the larvae had weaker motility, slow crawling, and a significantly reduced number of crawling traces after feeding on *S. marcescens*. There was no significant difference in the motility of housefly larvae between the SMPa/SMPb and Wa groups.

### Comparative analysis among intestinal microbiota of four groups of housefly larvae

To determine the impact of large-scale expansion of *S. marcescens* and phage SMP on the composition of the bacterial community, 12 intestinal samples of housefly larvae in the SM, SMPa/SMPb, and Wa groups were collected on the fourth day of feeding, and 16S rRNA sequencing was performed (BioProject ID: PRJNA881774). The results showed that a total of 716,662 high-quality bacterial sequences were produced, with sequence numbers 43040–88932; these sequences were standardized and grouped into 1616 operational taxonomic units (OTUs), and all samples were 97% similar (Additional file 4: Table S3).

**Fig. 5:**
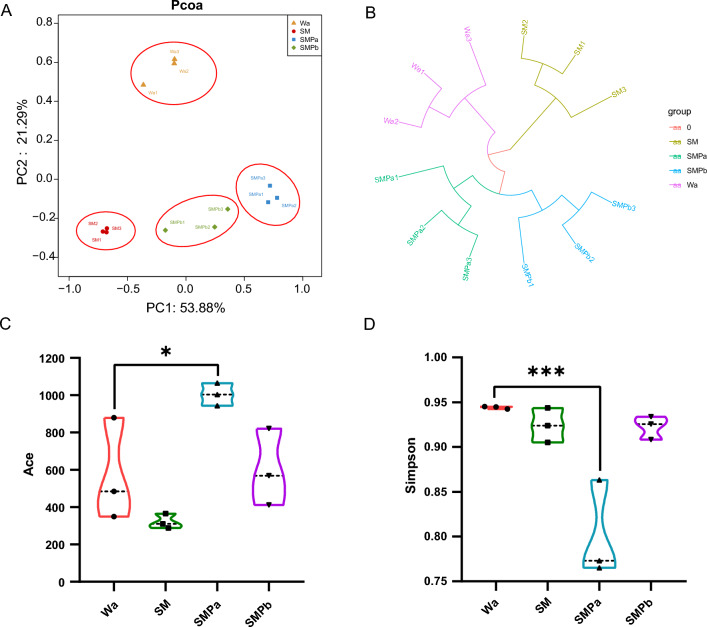
**A** Principal coordinate analysis of the gut bacteria in housefly larvae fed different diets. **B** A hierarchical clustering analysis for OTUs of all analyzed samples using unweighted pair group method with arithmetic mean (UPGMA). The alpha diversity of the gut microbiota based on Ace **C** and Simpson **D** indices for different groups. Data were compared by one-way ANOVA. The Brown–Forsythe test was used to assess the significance of the data. Values are the means  ±  standard deviations from triplicates of each treatment. *P < 0.05, **P < 0.01, ***P < 0.001

The addition of *S. marcescens* and phages affected the composition of the bacterial community (Fig. 5A, B, and Additional file 5: Table S4). Principal coordinate analysis (PCoA) (Fig. 5A) indicated that housefly larvae fed *S. marcescens* and phages differed in terms of β-diversity from housefly larvae in the Wa group. Moreover, Ace and Simpson indices showed that the bacterial diversity was altered in the SMPa group (*P* < 0.05; Fig. 5C, D).

**Fig. 6:**
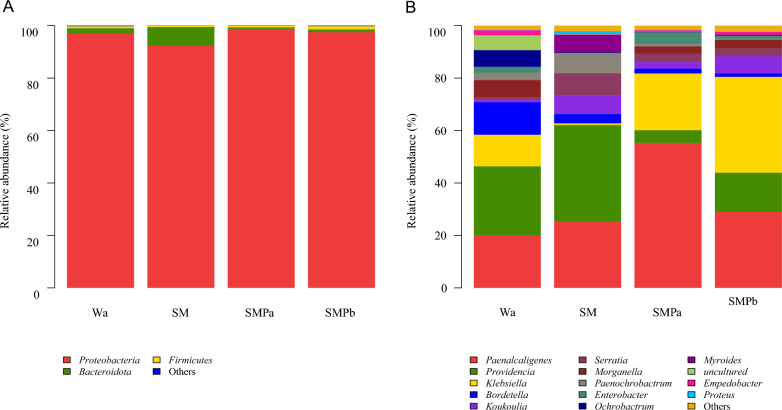
Relative abundance of the top three phyla and top 14 genera of gut bacteria in different treatments on the fourth day. **A** Gut microbiota composition at the phylum level. **B** Genus-level diversity of gut microbiota in the four experimental groups. The dominant bacterial genera based on OTUs identified in samples from each housefly larvae are shown

The analysis of dominant phyla and genera showed that larval samples subjected to the different treatments had only slight differences at the phylum level, but there were different bacterial community structures at the genus level (Fig. 6A, B). Among the 14 genera in the gut of larval samples (Fig. 6B), *Providencia* was the most abundant genus (26.5%) in the intestine of Wa group housefly larvae, whereas the SM group had a higher abundance of *Serratia* (8.14%) and *Providencia* (36.9%) and a notably lower abundance of *Klebsiella* (0.61%) and *Enterobacter* (0.11%). In contrast, we observed greater proportions of *Klebsiella* (21.66%) and *Enterobacter* (4.31%) in the SMPa group, while *Providencia* (4.94%) was less abundant.

### Network of gut microbiota in larvae exposed to different treatments

**Fig. 7:**
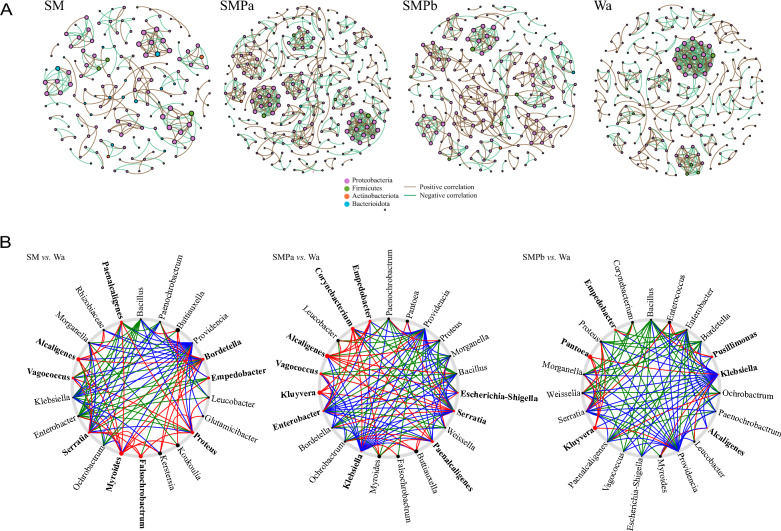
Intestinal bacterial co-occurrence microbiome networks of the different groups. **A** Effects of *S. marcescens* and phages on bacterial interaction networks. Each point in the graph represents a species, and related species are connected by a line. Each node represents a species, and related species are connected by a line. Red lines represent positive correlations, whereas green lines represent negative correlations. The node colors represent taxon classifications at the phylum level. **B** Based on a NetShift analysis of the Wa, SM, SMPa, and SMPb groups are marked SM-Wa, SMPa-Wa, and SMPb-Wa. Red colored nodes indicate important driver taxa

To analyze bacterial correlations within bacterial communities, we constructed interaction networks. The interaction between the gut microflora of housefly larvae was substantially altered by feeding on *S. marcescens* and phage. Compared with the findings in the Wa group, in the gut of housefly larvae fed *S. marcescens*, the numbers of total nodes and total links, average degree, and average path length of the flora interaction network in housefly larvae fed *S. marcescens* decreased, while the SMPa group had more nodes and links, a higher average degree, and a longer average path length (Fig. 7A and Additional file 6: Table S5). We thus speculated that there was less contact between beneficial bacteria in the bacterially treated group and more contact between beneficial bacteria in the phage-treated group [39].

By performing NetShift analysis between the communities of the Wa, SM, and SMPa/SMPb groups, *Alcaligenes*, *Serratia*, *Proteus*, and *Bordetella* were identified as driver genera in the initial microbiomes of housefly larvae fed *S. marcescens*, whereas *Alcaligenes*, *Kluyvera*, *Enterobacter*, and *Klebsiella* were identified as driver genera in the initial microbiomes of housefly larvae fed phage.

### Potential impact of microbiota disturbance on the health of housefly larvae

**Fig. 8:**
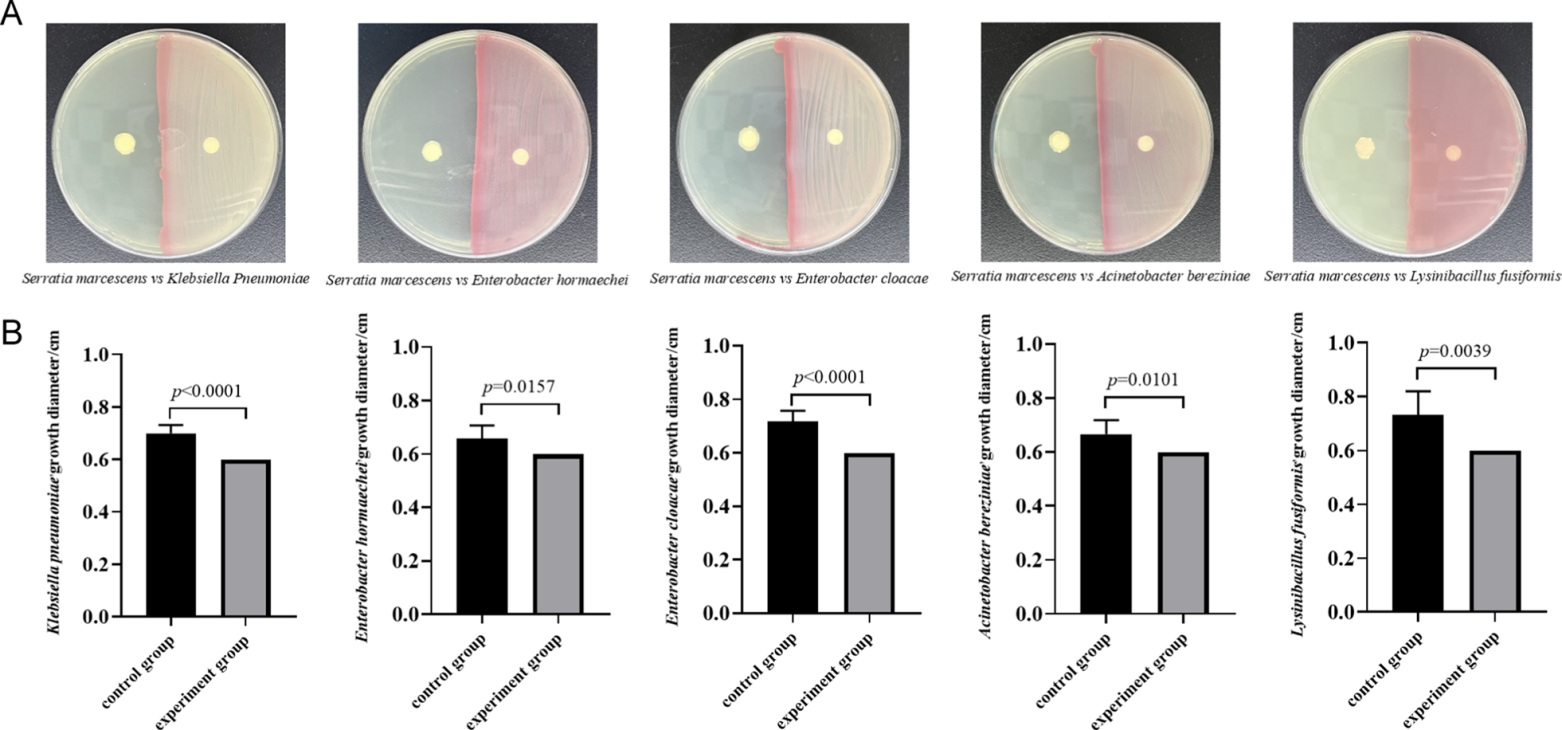
Antagonism experiment of *S. marcescens* and *K. pneumoniae*, *E. hormaechei*, *E. cloacae*, *A. bereziniae*, and *L. fusiformis* in the housefly larval intestine. **A** *Serratia marcescens* was seeded on the right side of the plate, while the opposite side was set as the control. A filter paper was dipped into *E. hormaechei*, *K. pneumoniae*, *E. cloacae*, *L. fusiformis*, and *A. bereziniae* cultures. **B** Competitive inhibition between *S. marcescens* and *K. pneumoniae*, *E. hormaechei*, *E. cloacae*, *A. bereziniae*, and *L. fusiformis* in the housefly larval intestine. Data are shown as the mean ± SEM. The *t*-test was used for statistical analysis

Bacterial invasion and phage predation induce disorders of intestinal bacteria. Indeed, in this study, there were major differences in the genus-level composition among the groups, such as in those of *Providencia*, *Klebsiella*, and *Enterobacter*. In particular, we found that the increase or decrease (phage predation) in *S. marcescens* had cascading effects on the bacterial communities. For example, after feeding on the phage targeting *S. marcescens*, observable shifts occurred in *Providencia*, *Klebsiella*, and other genera. To explore the interaction between these bacteria and *S. marcescens*, we conducted an antagonism assay (Fig. 8) with gut bacteria (*K. pneumoniae*, *E. hormaechei*, *E. cloacae*, *A. bereziniae*, and *L. fusiformis*) of housefly larvae. The results showed that *S. marcescens* inhibited the growth of *K. pneumonia*, *E. hormaechei*, *E. cloacae*, *A. bereziniae*, and *L. fusiformis*. In the laboratory, housefly larvae had previously been orally administered the cultivable bacterial strains *P. stuartii*, *P. vermicola*, *K. pneumonia*, *E. hormaechei*, and *E. cloacae*, confirming that *E. hormaechei* and *K. pneumoniae* are beneficial bacteria in the gut of housefly larvae and promoted their growth, while *Providencia*, as harmful bacteria, had lethal effects on the larvae. The above results show that the invasion of *S. marcescens* resulted in a change in the proportions of bacteria, as evidenced by a reduction in the abundance of beneficial bacteria and an increase in that of harmful bacteria, which together exert a negative effect on the health of housefly larvae. Conversely, a decrease in the proportion of *S. marcescens* upon targeting by phages resulted in an increase in the abundance of beneficial bacteria and a decrease in that of harmful bacteria, which promoted the growth and development of housefly larvae.

### Effects of oral intake of *S. marcescens* and phages on phenoloxidase activity in housefly larvae

**Fig. 9:**
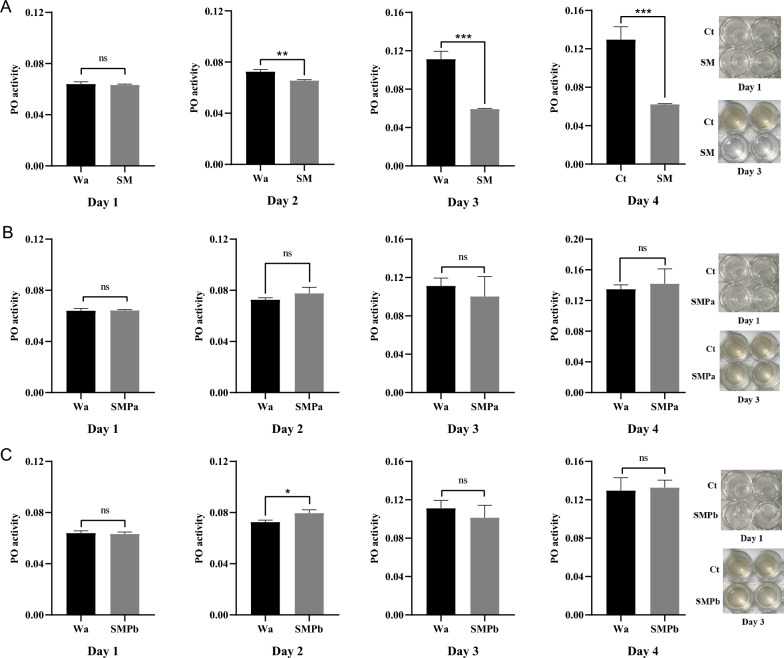
Changes in the phenoloxidase activity in the hemolymph of housefly larvae on days 1–4 after feeding on **A** *S. marcescens* and **B**, **C** phage. Data are shown as the mean ± SEM. The *t*-test was used for statistical analysis. *P < 0.05, **P < 0.01, ***P < 0.001

We analyzed the effects of bacteria and phages on phenoloxidase activity in the larval hemolymph to study the innate immunity of the larvae. The results showed that on the first day after oral administration of *S. marcescens* to housefly larvae, there was no difference in the phenoloxidase activity among the groups; however, on the second, third, and fourth days, when compared with the findings in the Wa group, only the SM group exhibited no melanization, and the phenoloxidase activity in hemolymph was significantly inhibited (Fig. 9).

## Discussion

The intestinal microbiome plays pivotal roles in insect hosts, including in their growth, adaptation, and reproduction [40, 41]. Infection with or purging of the target bacteria is often used to study the effect of symbiotic bacteria on host insects [42]. The *Serratia* genus is a bacterial taxon that is widespread in insects and is pathogenic to various insect groups [43–45]. However, to date, only a few studies have focused on the pathogenic mechanism underlying the effects of *S. marcescens* on housefly larvae. The interactions between *S. marcescens* and the diversity of the intestinal bacterial community have also remained unclear so far.

In this study, the effect of phage targeting on the growth and development of larvae was determined by measuring changes in the abundance of *S. marcescens*. Our results showed that *S. marcescens* not only disturbed the composition of the gut bacterial community and suppressed the growth of beneficial bacteria but also notably disrupted intestinal tissue, as revealed by trypan blue staining. Moreover, it inhibited the growth of housefly larvae. The phage not only knocked down its bacterial targets but also indirectly enriched the bacterial community colonizing the housefly larval gut through cascading effects. The addition of bacteriophages to the feed of houseflies promoted the proliferation of bacteriophages in wheat bran on the one hand and removed *S. marcescens* from the feed on the other hand (Additional file 7: Fig. S2).

Compared with the findings in the control group, feeding on *S. marcescens* substantially affected the commensal bacteria of housefly larvae at the genus level. Among them, *Providencia*, as bacteria associated with a high mortality rate in flies, always remained at a high level [46]. Research has shown that *P. stuartii* and *P. vermicola*, which are “harmful bacteria” in housefly larvae, can significantly inhibit the growth and development of these larvae [5], proving that *Providencia* plays a substantial role in the death of houseflies. In contrast, we found that the abundance of *Enterobacteria* and *Klebsiella* in the gut of housefly larvae was significantly reduced. *Klebsiella* is a genus of the *Enterobacteriaceae* family that is widely distributed in the intestinal tract of insects and has been shown to significantly promote black soldier fly development [47]. It has also been reported that *Klebsiella oxytoca* can be used as a probiotic to improve the mating success rate and viability of *B. dorsalis* flies [48]. Meanwhile, the oral intake of the beneficial bacterium *E. hormaechei* by housefly larvae was shown to increase their growth and development and improve their humoral immunity [5, 9]. Additionally, under aseptic conditions, *E. cloacae* inoculation was found to provide additional protection against infection, which improved fitness [49]. From the in-depth understanding of microbial networks obtained in this study, we presented the possibility that *S. marcescens* induced the overgrowth of some pathogenic strains while acting against beneficial bacteria through niche competition and nutrient limitation. To fully confirm this competitive mechanism, we performed a plate confrontation assay, with the results demonstrating that *S. marcescens* significantly inhibited the growth of *K. pneumoniae*, *E. hormaechei*, *E. cloacae*, *A. bereziniae*, and *L. fusiformis.* Therefore, the invasion of *S. marcescens* changed the interaction between the members of gut microbial communities and reduced the viability of housefly larvae.

Our findings also suggested that the model of phage amplification established by the single administration of phages could be leveraged as a powerful tool to knock down *S. marcescens* and change the microbiome. To date, most studies have focused on phages shaping the gut flora in mammals [50–53], but our understanding of various studies raises an intriguing question of how these phages mediate the abundance and composition of host-associated bacteria in the insect gut ecosystem. The phage predation experiment of *Serratia marcescens* *S. marcescens* in our study showed changes in the intestinal flora composition and co-occurrence network. We found that the abundance of *Providencia* decreased, whereas that of *Serratia*, *Enterobacter*, and *Klebsiella* increased in the phage-amplified samples of housefly larvae. This may be attributed to the resistance of *S. marcescens* to phages targeting the bacteria after 4 days of feeding. The phage SMP has a narrow intraspecies host range and had no apparent impact on other gut bacteria (*P. stuartii*, *P. vermicola*, *K. pneumonia*, *E. hormaechei*, *E. cloacae*, *A. bereziniae*, *L. fusiformis*, *P. aeruginosa*, *L. lactis*, and *B. safensis*) of housefly larvae. Therefore, the changes in species (*Klebsiella*, *Enterobacter*, and *Providencia)* not susceptible to the phage were driven indirectly by phage predation. We speculate that a decrease in the proportion of *S. marcescens* has a reduced inhibitory effect on *K. pneumoniae*, *E. hormaechei*, and *E. cloacae* and provides more space and nutrients for other microorganisms [54]. Moreover, a decrease in the proportion of *S. marcescens* in wheat bran provides a healthier diet for housefly larvae. Taken together, these results suggest that a lack of symbiotic bacteria may be an important factor in insect health, possibly through increasing interactions with harmful bacteria.

## Conclusions

The introduction or loss (phage predation) of certain gut bacteria can significantly change the composition of the bacterial community in the insect gut, thereby affecting host development. The results of this study revealed the important role of the intestinal flora of housefly larvae after *S. marcescens* infection and provided a new avenue for further studies on the pathogenic mechanisms of *S. marcescens* in housefly larvae. Our findings also revealed that bacterial phages could be used as specific bacterial control tools for investigating the pathogenic mechanism of *S. marcescens* based on the dynamic changes and bacterial interactions of host gut microbiota. By highlighting the details of the dynamic diversity and variation in gut bacterial communities, this study illustrates the essential relationship between gut microbiota and larval development when housefly larvae encounter pathogenic bacteria, thereby providing a framework to guide future investigations on insect–phage–bacteria–host relationships and models of their interactions.

## Supplementary Information

**Additional file 1: Table S1.** Name, location, year of isolation, and morphology of the phage used in this study.

**Additional file 2: Table S2.** Infectivity range of phage SMP against gut bacteria of housefly larva. Phages were spotted onto the lawns of each bacteria and incubated overnight at 37 °C. Zones of clearing indicated infectivity.= lysis;= no lysis.

**Additional file 3: Figure S1.** Annotated genome maps for the phage SMP. In the circular genome map, the outermost black circle represents the full length of the genome, the innermost multicolored circle represents annotated functional proteins, the second outermost blue circle represents the GC skew, and the third outermost purple circle represents the GC skew content.

**Additional file 4: Table S3.** Information derived from 16S rRNA gene sequencing.

**Additional file 5: Table S4.** PCoA score for each sample.

**Additional file 6: Table S5.** Topological properties of bacterial co-occurrence networks associated with the different treatments.

**Additional file 7: Figure S2.** Phage targeted reduction of *S. marcescens* in wheat bran. Ct, SMPa, and SMPb represent wheat bran samples treated with sterile water and sterile water containing 10^7^ and 10^11^ PFU/mL phage, respectively. Values are the means ± standard deviations from triplicates of each treatment. *P < 0.05, **P < 0.01, ***P < 0.001, ****P < 0.0001. n.s., no significance.

## Data Availability

Phage sequences were deposited in the NCBI (SMP: OP490597). The 16S rRNA gene sequence data of the microbiome were stored in the SRA database (BioProject ID: PRJNA881774).

## Abbreviations

16S rRNA: 16S ribosomal RNA
OTU: Operational taxonomic units
