# Supplementary material for: Serratia marcescens in the intestine of housefly larvae inhibits host growth by interfering with gut microbiota
Source: Parasit Vectors. 2023 Jun 10;16:196. doi: 10.1186/s13071-023-05781-6 (PMC10257315; doi:10.1186/s13071-023-05781-6)
Supplement: Supplementary file 1 — Additional file 1: Table S1. Name, location, year of isolation, and morphology of the phage used in this study. [file 13071_2023_5781_MOESM1_ESM.pdf]

Supplementary materials

Table S1 Table showing the name,location,year of isolation and morphology of the phage used in this study.

Name	Location and year of isolation	Morphology
SMP	Taian China （36° 12′ 26″ E,117°6′4″ N）<br>2022	*Caudovirales*（*order)*<br>*Myoviridae*（*family*）
