# Supplementary material for: Serratia marcescens in the intestine of housefly larvae inhibits host growth by interfering with gut microbiota
Source: Parasit Vectors. 2023 Jun 10;16:196. doi: 10.1186/s13071-023-05781-6 (PMC10257315; doi:10.1186/s13071-023-05781-6)
Supplement: Supplementary file 2 — Additional file 2: Table S2. Infectivity range of phage SMP against gut bacteria of housefly larva. Phages were spotted onto the lawns of each bacteria and incubated overnight at 37 °C. Zones of clearing indicated infectivity.= lysis;= no lysis. [file 13071_2023_5781_MOESM2_ESM.pdf]

**Table S2** Infectivity range of phages SMP against gut bacteria of housefly larva. Phages were spotted onto lawns of each bacteria and incubated overnight at 37°C. Zones of clearing indicated infectivity. (+) = lysis; (-) = no lysis

| **Bacteria** | **phage SMP** |
|---|---|
| *S. marcescens* | + |
| *P. stuartii* | - |
| *P. vermicola* | - |
| *K. pneumonia* | - |
| *E. hormaechei* | - |
| *E. cloacae* | - |
| *A. berezinia* | - |
| *L. fusiformis* | - |
| *P. aeruginosa* | - |
| *L. lactis* | - |
| *B. safensis* | - |
