# Supplementary material for: Serratia marcescens in the intestine of housefly larvae inhibits host growth by interfering with gut microbiota
Source: Parasit Vectors. 2023 Jun 10;16:196. doi: 10.1186/s13071-023-05781-6 (PMC10257315; doi:10.1186/s13071-023-05781-6)
Supplement: Supplementary file 3 — Additional file 3: Figure S1. Annotated genome maps for the phage SMP. In the circular genome map, the outermost black circle represents the full length of the genome, the innermost multicolored circle represents annotated functional proteins, the second outermost blue circle represents the GC skew, and the third outermost purple circle represents the GC skew content. [file 13071_2023_5781_MOESM3_ESM.pdf]

**Table S5** Topological properties of bacterial co-occurrence networks associated with the different treatments.

| Group | Network indices | | | | | | |
|---|---|---|---|---|---|---|---|
| | **Total nodes** | **Total links** | **Average degree** | **Average clustering coefficient** | **Average path distance** | **Positive correlation** | **Negative correlation** |
| **Wa** | 229 | 470 | 4.105 | 0.845 | 1.818 | 56.81% | 43.19 |
| **SM** | 163 | 244 | 2.994 | 0.845 | 1.409 | 57.79% | 42.21 |
| **SMPa** | 265 | 598 | 4.513 | 0.856 | 2.601 | 57.69% | 42.31 |
| **SMPb** | 212 | 413 | 3.896 | 0.812 | 2.706 | 67.55% | 32.45 |
