# Supplementary material for: Serratia marcescens in the intestine of housefly larvae inhibits host growth by interfering with gut microbiota
Source: Parasit Vectors. 2023 Jun 10;16:196. doi: 10.1186/s13071-023-05781-6 (PMC10257315; doi:10.1186/s13071-023-05781-6)
Supplement: Supplementary file 5 — Additional file 5: Table S4. PCoA score for each sample. [file 13071_2023_5781_MOESM5_ESM.pdf]

Table S3 Information derived from the 16S rRNA gene analysis in this study. Data are expressed as the mean ± standard deviation of three replicate samples in each sampling.

Samples		Clean Reads	OTU number	Shannon	Simpson	Chao1	Ace	Coverage
	**Wa1**	51628						
**Wa**	**Wa2**	44833	444.67±126.04	5.05±0.13	0.9440±0.0014	570.05±293.16	571.18±275.16	0.9978±0.0016
	**Wa3**	68354						
	**SM1**	48839						
**SM**	**SM2**	46550	298.33±36.50	4.73±0.35	0.9242±0.0193	319.48±34.01	321.37±39.85	0.9990±0.0005
	**SM3**	46120						
	**SMPa1**	82208						
**SMPa**	**SMPa2**	82356	675±51.26	4.12±0.45	0.8005±0.0545	1020.51±60.15	1004.45±61.03	0.9966±0.0004
	**SMPa3**	88932						
	**SMPb1**	43040						
**SMPb**	**SMPb2**	49014	468.67±101.03	4.96±0.17	0.9225±0.0131	598.55±215.50	600.30±206.98	0.9973±0.0004
	**SMPb3**	64788						
