# Supplementary material for: Serratia marcescens in the intestine of housefly larvae inhibits host growth by interfering with gut microbiota
Source: Parasit Vectors. 2023 Jun 10;16:196. doi: 10.1186/s13071-023-05781-6 (PMC10257315; doi:10.1186/s13071-023-05781-6)
Supplement: Supplementary file 6 — Additional file 6: Table S5. Topological properties of bacterial co-occurrence networks associated with the different treatments. [file 13071_2023_5781_MOESM6_ESM.pdf]

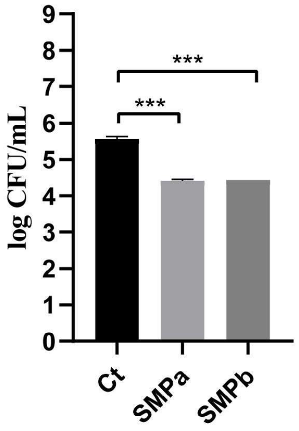

Fig. S2. Phage targeted reduction of *S. marcescens* in wheat bran. Ct, SMPa and SMPb represent wheat bran samples treated with sterile water and sterile water containing 10^7 and 10^{11} PFU/ml phage, respectively. Data are shown as the mean ± standard deviations. Number of bacteria among different treatment groups were compared by using one-way ANOVA followed by Tukey test.Each treatment included 5 biological replicates. *$P < 0.05$, **$P < 0.01$, ***$P < 0.001$, ****$P < 0.0001$. n.s., no significance.
