# Supplementary material for: Serratia marcescens in the intestine of housefly larvae inhibits host growth by interfering with gut microbiota
Source: Parasit Vectors. 2023 Jun 10;16:196. doi: 10.1186/s13071-023-05781-6 (PMC10257315; doi:10.1186/s13071-023-05781-6)
Supplement: Supplementary file 7 — Additional file 7: Figure S2. Phage targeted reduction of S. marcescens in wheat bran. Ct, SMPa, and SMPb represent wheat bran samples treated with sterile water and sterile water containing 107 and 1011 PFU/mL phage, respectively. Values are the means ± standard deviations from triplicates of each treatment. *P < 0.05, **P < 0.01, ***P < 0.001, ****P < 0.0001. n.s., no significance. [file 13071_2023_5781_MOESM7_ESM.pdf]

**Table S4** The PCoA score for each sample.

| Group | PCoA score | |
|---|---|---|
| | **PC1** | **PC2** |
| Wa1 | -0.364328759 | 0.484066423 |
| Wa2 | -0.100337162 | 0.593995489 |
| Wa3 | -0.097660064 | 0.615797769 |
| SM1 | -0.679813861 | -0.271331555 |
| SM2 | -0.711312931 | -0.267744627 |
| SM3 | -0.676442268 | -0.251447012 |
| SMPa1 | 0.703389265 | -0.117260518 |
| SMPa2 | 0.77980127 | -0.095006338 |
| SMPa3 | 0.719161446 | -0.032673372 |
| SMPb1 | -0.175901281 | -0.261096031 |
| SMPb2 | 0.246014776 | -0.244549585 |
| SMPb3 | 0.35742957 | -0.152750642 |
